# Implementation challenges and opportunities for improved mass treatment uptake for lymphatic filariasis elimination: Perceptions and experiences of community drug distributors of coastal Kenya

**DOI:** 10.1371/journal.pntd.0009012

**Published:** 2020-12-28

**Authors:** Doris W. Njomo, Bridget W. Kimani, Lydiah W. Kibe, Collins Okoyo, Wyckliff P. Omondi, Hadley M. Sultani

**Affiliations:** 1 Eastern and Southern Africa Centre of International Parasite Control (ESACIPAC) Kenya Medical Research Institute (KEMRI), Nairobi, Kenya; 2 Division of Vector Borne and Neglected Tropical Diseases, Ministry of Health, Nairobi, Kenya; University of Buea, CAMEROON

## Abstract

Community drug distributors (CDDs) who are volunteers have the responsibility of awareness creation, household census, drug distribution and record-keeping and are thus key stakeholders in the campaign for Lymphatic Filariasis (LF) elimination. Taking into account their experiences and perceptions is important for a successful elimination campaign. We conducted a qualitative study in 2018 to identify implementation challenges and opportunities for improved mass drug administration (MDA) uptake based on the CDDs perceptions and experiences. Within a larger study that used mixed methods quasi-experimental design, we collected qualitative data from two wards in Kaloleni Sub-County of Kilifi County which was purposively selected owing to its low, 56% and 50.5% treatment coverage in 2015 and 2016 respectively. Focus group discussions (FGDs) (n = 8) and in-depth interviews (IDIs) (n = 8) with CDDs, IDIs (n = 22) with opinion leaders and IDIs (n = 8) with health workers were conducted and the data analyzed by QSR NVIVO version 10 according to thematic areas. The results showed that based on the perceptions and experiences of the CDDs, several challenges: communities’ refusal to take the drugs; absenteeism during MDA; non-adherence to CDDs selection criteria; inadequacy in number of CDDs engaged during the campaign and training provided; insufficiency of drugs issued to CDDs; lack of CDDs supervision and low motivation negatively impact on MDA uptake. Opportunities to address the challenges included: awareness creation on MDA, health education on LF and observation of hygiene during drug administration, increased duration of awareness creation and drug administration, adherence to CDDs selection criteria and putting into consideration the vastness of an area and population density while deploying CDDs. Other opportunities include: improved CDDs training and scheduling; issuing of enough drugs to CDDs to meet the communities’ demand and improved supervision and motivation of CDDs. Addressing the challenges highlighted is an important step of maximizing MDA uptake. The opportunities presented need to be considered by the NTD program personnel, the county health personnel and the community while planning the implementation of MDA campaigns.

## Author summary

Elephantiasis infection which is common in tropical areas is targeted for elimination globally through delivery of drugs to all populations living in areas where infections occur. Community Drug Distributors (CDDs) are persons who live in the affected communities and who volunteer themselves to distribute drugs after receiving training from the health care workers. Understanding the challenges and opportunities for improved community drug uptake from the perspectives and experiences of the CDDs is important for a successful elimination campaign.

As part of a larger study to address barriers of drug uptake in Kenya, we collected qualitative data to explore the challenges and opportunities for improved drug uptake in two wards of Kilifi County. The data was collected through focus group discussions and in-depth interviews and analyzed.

For drug uptake to be improved, the CDDs’ mentioned challenges related to how their selection is done, number of CDDs engaged, quality of their training, quantities of drugs issued to them, supervision and motivation that need to be addressed. The communities targeted for treatment need well scheduled health education activities on elephantiasis, awareness creation on drug distribution and drug reaction for them to understand the importance of receiving the treatment. These findings suggest the need for strong collaborations between NTD program, county health personnel and the community that are useful in the planning the implementation of successful national programs.

## Introduction

The Global Program to Eliminate Lymphatic Filariasis (GPELF) which was launched in the year 2000 has two principal goals: (i) to interrupt LF transmission and (ii) to manage morbidity and prevent disability in endemic countries [1]. For LF transmission to be interrupted, the GPELF recommends annual community-wide mass drug administration (MDA) of antifilarial tablets; diethylcarbamazine citrate (DEC) or ivermecticin and albendazole to entire at-risk populations [2,3]. Recent scientific studies, led by the Death of Onchocerciasis and Lymphatic Filariasis (DOLF) project at Washington University in St. Louis, found that a three-drug regimen, using all three of the medicines typically delivered as a standard two-drug regimen to prevent LF (ivermectin + albendazole or diethylcarbamazine + albendazole (IDA)), is dramatically more effective for achieving sustained clearance of microfilariae from infected persons [4]. World Health Organization conducted a rigorous review of data from safety and efficacy trials of the triple drug regimen and in November 2017 provided updated treatment guidelines that endorsed the use of IDA as an alternative MDA regimen for LF elimination programmes [5]. At the start of GPELF, 81 countries were considered to be LF endemic and a review of epidemiological data confirmed that 10 countries did not require preventive chemotherapy [6]. From 2000 to 2018, 7.7 billion treatments were delivered to more than 910 million people at least once in 68 countries, considerably reducing transmission and causing a decline of the population requiring MDA by 42% (597 million) where infection prevalence has been reduced below elimination thresholds [7].

In Kenya, LF is endemic in 6 counties of the coastal region where about 4 million people are at risk of infection [8]. The LF elimination program was initiated in the year 2002 but could not be sustained due to administrative and financial challenges. In 2015 the program was re-started and has since achieved 100% geographical coverage. Sentinel surveys conducted throughout the coastal region in 2015 and 2016 showed an overall prevalence in circulating filarial antigen (CFA) positive persons of 1.3% with 2 counties namely Kilifi and Kwale having a prevalence < 1.7% [9]. Lamu East and Jomvu sub-counties were identified as the sites with the highest prevalence of LF (6.3% and 6.7% antigenemia, respectively) [9]. Due to the historically high prevalence of LF, the intermittent history of MDA and the sub-optimal coverage, both Lamu counties and Jomvu sub-county were selected to receive the WHO-approved triple drug (IDA) therapy to accelerate LF elimination.

The Kenyan health system through the Community Health Strategy outlines the role of Community Health Volunteers (CHVs) who are the primary health care providers at the community level known as level one [10]. The Strategy further describes the role of the Community Health Extension Workers (CHEWs) who are in charge of community units comprising of 250 households of approximately 1000 persons of supervising the CHVs working under them. It is the CHVs who conduct drug distribution in the Kenyan LF program and are selected based on the required numbers and assume the title of Community Drug Distributors (CDDs). The selection process is designed in such a way that community leaders through open meetings at community level sensitize community members and together they select CDDs. The selection criteria include: ability to read and write; keep records; trustworthiness; well known by the community members; and willingness to distribute drugs to all eligible persons in allocated areas [11]. Training of the CDDs is done by the CHEWs and is planned to take one full day [12]. The training provides general information on LF, community mobilization and registration, planning and implementation of MDA, and completion of treatment registers. The training includes both theory and practical sessions on how to conduct household registration and fill in the tally sheets. The CDDs who are essentially volunteers from the community are charged with undertaking the role of household census, community sensitization, drug distribution and record-keeping after receiving training from the health personnel [12]. Community mobilization and sensitization is conducted through banners posted in public places, pamphlets distributed to each household, chiefs’ meetings, and door-to-door visits by the CDDs.

The distribution of drugs is done house-to-house to all persons aged two years and above. The whole exercise takes five days: two days’ registration and three days’ drug administration including mop-ups i.e. revisits to those missed out on initial days. The CDDs are given program monetary incentives amounting to Kenyan Shillings 500 (5 USD) for each day which is consistent with nationally-recognized daily subsistence allowance rates. During the distribution exercise, the CHEWs are expected to be on standby to supervise the CDDs and manage any side effects.

Some factors reported to have positive influence on the CDDs’ role include; desire to help prevent their communities from getting LF infection, recognition and experience in educating their community members [13,14]. Factors such as short training duration less than the recommended full day have been reported to reduce the CDDs motivation and to compromise the quality of their services thus contributing negatively on their confidence in conducting community sensitization [15]. Krentel et *al*., (2018) reiterates the urgent need to conduct implementation research to find out the challenges related to the CDDs and to use the information we get to begin constructive dialogue with health personnel to understand the pertinent issues and to craft feasible solutions [16]. The current study which was conducted in Kaloleni sub-county in Kilifi county sought to identify some of the implementation challenges and opportunities for improved MDA uptake from the perspectives and experiences of the CDDs.

## Methods

### Ethics statement

Ethical clearance was received from the Kenya Medical Research Institute (KEMRI), Scientific and Ethics Review Unit (SERU No. 3666) and written informed consent sought from all the study participants. An information sheet was provided to all individuals who were 18 years and above invited to participate in the study in Swahili. Participants underwent written informed consent and agreed to have the IDIs and the FGDs audio-recorded. During data capture and transcription, participant names were replaced with alphanumeric unique identifiers to ensure anonymity and confidentiality. The following are examples of unique identifiers used: CDD-FGD-03-Kaloleni which indicates FGD number 3 conducted with CDDs in Kaloleni Ward; HW-IDI-01-Kayafungo indicates Health workers’ IDI number 1 conducted in Kayafungo Ward and OL-IDI-02- Kaloleni for Opinion leaders’ IDI number 2 conducted in Kaloleni Ward.

### Study area

Administratively, Kilifi County is made up of 7 sub-counties: Kilifi North, Kilifi South, Kaloleni, Rabai, Ganze, Malindi and Magarini. The current study was conducted in Kaloleni Sub-County which has a population of 159, 739 [17]. Two wards i.e. Kaloleni with a population of 41, 689 of which an average of 36.8% is urban [17] and Kayafungo with a population of 22, 250 people of different ethnic background formed the rural study area. Farming and business are the main economic activities of the area.

### Study design and setting

Based on the MDA program data from MOH, Kenya, Kaloleni sub-county was purposively selected owing to its low, 56% and 50.5% treatment coverage in 2015 and 2016 respectively. Kaloleni and Kayafungo wards were then selected purposively for the study. In 2015, Kaloleni ward had a treatment coverage of 58% and Kayafungo, 54% and in 2016, Kaloleni had 62% and Kayafungo 39% all below the recommended minimum treatment coverage of 65%. We collected qualitative data in 2018 while conducting a larger mixed methods quasi-experimental study in the two wards. The data being presented in the current paper was collected through FGDs and in-depth interviews (IDIs) with CDDs, separate IDIs that were conducted with opinion leaders and with health workers.

### Participant selection

A total of 6 villages in Kaloleni and Kayafungo wards that were considered to have experienced substantial implementation challenges were purposively selected with the support of the area chiefs. The selected villages were Town Center and Vishakani in Kaloleni ward and Mirihi ya Kirao, Gogoraruhe, Gandini A and Gandini B in Kayafungo ward. The CDDs who distributed the anti-filarial drugs in the study villages during the 2017 and 2018 MDA participated in the study. The opinion leaders included the community groups leaders- political, social and religious groups while the health workers were the CHEWs designated as CDDs’ supervisors during the 2018 LF MDA campaign. A total of 8 FGDs (S1 Text) and 8 IDIs (S2 Text) were conducted with CDDs, 22 IDIs (S3 Text) with opinion leaders and 15 IDIs (S4 Text) with health workers. All the study participants were purposively selected and the number of FGDs and IDIs was determined by the level of saturation.

### Data collection

The data collection was moderated by 3 social scientists from KEMRI assisted by 8 trained field assistants with the aid of interview guides using Swahili, the local language. The design was iterative and there was a back and forth process which included data collection and analysis and further sample selection therefore giving early insights and influencing selection of more participants up to the point where no newer information was being gathered. Standard procedures including maintaining a neutral stance, probing and allowing the respondents to express themselves without asking leading questions, asking general questions before specific questions and varying questions wording to avoid seeming repetitive were adhered to [18]. Each FGD and each IDI took a minimum of 40 minutes to a maximum of 60 minutes and both were held in private areas to ensure participants’ confidentiality. Notes were taken during the data collection process and voice recorders used to record all the information in the local language. Challenges experienced by the CDDs during MDA campaign and possible mitigating measures were identified based on the CDDs perspectives and experiences.

### Data management

The recorded data were coded and later transcribed and translated into English. Double transcription and translation and back translation was done among the investigators so as to agree on the meaning of the transcripts and minimize bias. The hard copies of the data were stored in lockable and secure cabinets. To ensure quality control, the soft copies were stored in computers with passwords, with authorized access by the Principal Investigator to the study investigators.

### Data analysis

The data were coded and entered into QSR NVIVO version 10 for management and analysis. Manual analysis was further conducted according to study themes which were determined prior to the analyses. A code sheet was created following the IDIs and FGDs guides after which, the textual data was coded into selected themes and a master sheet analysis was carried out, giving all the responses a theme. Thematic analysis was used where responses were categorized into themes and then ideas formulated by looking at the patterns of responses [19]. The analyzed data were presented in text form. Representative quotes were embedded within the results to illustrate themes, with minor grammatical alterations to improve readability. The pre-determined themes based on challenges faced included: community members’ refusal to take the drugs; community members’ absenteeism during MDA; non-adherence to CDDs selection criteria and insufficiency of number of CDDs engaged in comparison to the workload, inadequacy of CDDs training; insufficiency of drugs issued to CDDs, lack of CDDs supervision and low motivation.

## Results

### Background characteristics of the study participants

#### The community drug distributors’ FGDs

In total, eight FGDs with a total of 74 CDDs who had distributed anti-filarial drugs in the six villages during November 2017 MDA were conducted. A large majority, (97.3%) had attained primary school level of education and above and less than a half (45.1%) were in the age category of 18–39 years of age. The socio-demographic characteristics of the CDDs are presented in Table 1.

**Table 1:** Socio-demographic Characteristics of the Study Participants CDDs FGDs.

Description CDDs	Frequency (n = 74)	Percentage %
**Gender**		
Male	21	**28.4**
Female	53	**71.6**
**Age in years**		
18–29	3	**4.1**
30–39	30	**41**
40–49	26	**35**
50–59	12	**16.2**
60–69	1	**1.4**
70–79	1	**1.4**
Missing	1	**1.4**
**Education Level**		
Never Attended School	2	**2.7**
Completed primary school but did not complete secondary school	39	**52.7**
Completed Secondary School	29	**39.2**
Further Studies after Secondary School	4	**5.4**
**Religion**		
Christian	62	**83.8**
Muslim	12	**16.2**
**Main Occupation**		
Farmer	40	**54.1**
Small business	19	**25.7**
Salaried worker	4	**5.4**
Casual laborer	3	**4.1**
Others, specify	8	**10.8**

#### Community drug distributors’ IDIs

Eight IDIs were conducted with CDDs who distributed drugs during the November 2018 MDA. One half, four were female and the other four, male. Four CDDs were farmers and another four, small business owners. A majority, five were Christians and all had attained primary school level of education. The youngest CDD interviewed was 36 years old and the oldest 52.

#### The opinion leaders

A total of 22 opinion leaders participated in the IDIs. A majority, (n = 19) were male and their age range was 25–73 years. Less than a half (n = 10) were farmers/small business owners, 12 were Christians and 10, Muslims and 13 of them had attained secondary school level or higher education.

#### The health workers

A total of 15 health workers whose designation is CHEW participated in separate IDIs. About a half (n = 8) were male. Three fifths, nine were Christians and the remaining, 6 were Muslims.

Table 2 presents the issues identified by CDDs that posed challenges to implementation of MDA and opportunities to address such changes from the CDDs’ experiences and perspectives. Results of the data from the opinion leaders and health workers are then presented to support the views of the CDDs by the thematic areas.

**Table 2:** Implementation challenges and opportunities for improved uptake.

Issue	Challenges	Opportunities for Improved Uptake
Community members’ refusal to take the drugs	Lack of perceived need for treatment Unaware of MDA Fear of Severe Adverse Events (SAEs) (abdominal pain, nausea and dizziness) Myths and misconceptions about LF disease Lack of hygiene in drugs handling	Awareness creation on LF, drugs used and eligibility Aggressive and repeated awareness creation about MDA Awareness creation about potential SAEs which are resilient Repeated health promotion activities Use of plastic spoons while administering the drugs
Community members’ absenteeism during MDA	Seasonal migration-for agricultural activities Occupation (working and living away from home periodically) Unaware of MDA timing	Duration and timing of MDA to be scheduled in consideration of farming season Awareness creation Repeated messaging on MDA
Lack of transparency in the CDDs selection process	Unknown CDDs Aged CDDs who had never attended school Same CDDs all years	Select CDDs who belong to the villages allocated Select youthful CDDs with some level of schooling Give others (CHVs) a chance to participate
Inadequacy of number of CDDs in comparison to the area to be covered	Limited number of CDDs, limited time to respond to community members’ questions Large area to be covered Few CDDs allocated to vast areas with sparse population	Engage more CDDs Allocate enough CDDs to large areas Allocate enough CDDs to vast areas to allow them reach all households
Inadequate training of CDDs	No training at all Inadequate training-too brief	Train CDDs adequately Call CDDs for training in good time and train adequately
Insufficiency of drugs issued to CDDs	Inadequate numbers of drugs for all community members	Issue enough drugs to cater for those absent during registration
Lack of CDDs supervision and low motivation	Little or no supervision at all Low motivation	Adequate supervision of CDDs for moral support Appreciate and motivate CDDs

### Community members’ refusal to take drugs “Some are saying the drugs are for other purposes and that they will not take them”

The CDDs reported that they encountered refusals from some community members who declined to take the drugs stating that they were not sick, with others fearing the side effects. Beliefs and social factors were also reported to lead to some refusals as some household members claimed that they go to church for prayers when sick and did not rely on taking drugs.

A CDD in Kayafungo ward stated that:

“Some people refuse to take the drugs, they say they will wait to get infected and treat themselves. There is a man who questioned me that who told you that we are sick and we need your drugs. You want me to take the drugs and sleep yet I have a lot to do for the day, go away with the drugs.” (CDD-FGD-02-Kayafungo)

A CDD in Kaloleni ward further stated that:

*“*In my opinion, if they are informed first, when the drugs are brought, they will obviously accept them, they first get informed, then the drugs be brought because if the drugs are brought without information, the previous myths are still there.” (CDD- FGD-03-Kaloleni)

Reports from the IDIs with opinion leaders further stated that the communities’ knowledge of the diseases and of MDA was poor as the time given for awareness creation was inadequate and CDDs had to spend time convincing the community members on the importance of taking the drugs with community members saying that the drugs are for family planning.

An opinion leader from Kaloleni ward stated that:

“In my opinion, I think the information usually does not get to everyone. There are so many challenges in these rural areas because there are areas that are inaccessible by road and information doesn’t reach. Some areas are also far and cannot be reached by the village elders in a short time.” (OL-IDI-004-Kaloleni)

An opinion leader from Kayafungo ward also stated that:

“There is a bigger percentage almost half who were thinking when they are given the drugs they are for family planning. And family planning you know it’s a choice, so people were saying you were told to do family planning you refused that is why the government has come with these drugs so that when you take them you will not bear children.” (OL-IDI-001-Kayafungo)

Suggestions for improved community uptake included enhanced sensitization about MDA being done and the information about swollen limbs and swollen genitals getting to the people early with an extended period of awareness creation before the MDA activity. The CDDs further emphasized on the need to regularize health promotion activities which should not happen sporadically during the MDA campaign. A CDD in Kayafungo thus stated:

“Our community is very willing to participate/cooperate on anything directed to them but please before the next distribution is done allocate enough time to educate them on the drugs and the disease. Make them understand that it is for their own good” (CDD*-*FGD-003-Kayafungo)

The opinion leaders further emphasized on the importance of repeated awareness creation with clear information on eligibility criteria, side effects of drugs putting emphasis that these would be resilient. Support by the health officers and chiefs was recommended as a good way of building trust and ensuring that the community members understood about the side effects expected after consuming the drugs. An opinion leader from Kayafungo ward stated that:

“Create enough awareness, come to us several times to tell us about the drugs not just once. Even if am given the drugs today I will take them because I know their importance.” (OL-IDI-001-Kayafungo)

The CDDs also mentioned that the important role played by pamphlets with pictorials used when sensitizing the community and getting distributed to every household that helped as people changed their stand and accepted to take the drugs after seeing pictures of those affected. A CDD in Kaloleni stated that:

*“*Through the pictures of people suffering from swollen genitals, swelling breast, swelling legs, were shown to the community to inform them about the actual disease and how it is like. The CDDs went door-to-door passing the massage.” (CDDs- IDI-002-Kaloleni)

The important role of pictures on the pamphlets in sensitization was further echoed by a health worker.

*“*The reaction was positive because they had a chance to see the pictures of people with swollen limbs and genitals for them to make decisions.” (HW-IDI-001-Kaloleni)

Other community members were reported to have refused the drugs because they felt that the handling with bare hands was unhygienic. The CDDs requested for spoons to ensure hygiene in drug dispensing and argued that this could also minimize wastage through spillage. A CDD in Kaloleni stated that:

“We have a challenge because we are just being given the drugs. So a lot of drugs are going to waste because we have to use the lid, you start counting for the person then you find others falling Now go to distribute the drugs and then you use your hands, there are no spoons, others do not want.” (CDD-FGD-004-Kaloleni)

### Community members’ absenteeism during MDA “When you visit a household and find out that some people are not in”

The CDDs expressed the absence of household members during MDA as a challenge that they experienced which they related to inadequate awareness creation with reminders for community members to expect the CDDs for the drugs administration. Other reasons given as to why household members were absent during MDA was the perception that the drugs caused them to sleep and vomit as they are too strong and even cause swelling of the limbs and genitals. Seasonal migration or temporal relocation of community members to farms for agricultural activities was a factor that contributed to absenteeism during MDA. The best time of the year when people would be available to receive the drugs would be while they were not busy on their farms in the months of April and September. In this respect the CDDs felt that there was need for continuous sensitization using a variety of strategies.

Participants in an FGD with CDDs in Kaloleni further stated that:

“Migration, some people are available during registration period but relocate without notice.” (CDD-FGD-001-Kaloleni)

The health workers stated some reasons for communities’ failure to participate in the programme included migration during MDA days. A health worker stated that:

“Migration, as some agree during mobilization and sensitization are absent during the MDA days.*”* (HW- IDI-002-Kayafungo)

The opinion leaders mentioned that conducting the MDA on days when people are likely to be at home and not in their farms would be a way of reaching many community members. An opinion leader from Kaloleni stated that:

“The best time to conduct the MDA is when people are not working on their farms that is April and September.*”* (OL-IDI-003-Kaloleni)

A CDD in Kayafungo stated that:

“From my opinion I feel the period to be too little because you will find a situation where by the members of a particular house are not at home, and they go to another village ending up some people not being reached and the CDDs not coming back because of the big area they are supposed to cover.” (CDD-FGD-004- Kayafungo)

To tackle the issue of absenteeism due to migration, opinion leaders felt that the duration of the MDA should be extended especially in the rural areas and areas with high population density. An opinion leader in Kaloleni stated that:

*“*The period should be a little longer so that the CDDs can be able to revisit the houses where people were not present, that way everyone will be reached.” (OL-IDI-004-Kaloleni)

### Non-adherence to the CDDs’ selection process “Select CDDs who are literate and energetic”

The selection criteria of the CDDs was reported to not have been adhered to with CDDs who had never attended school, were of a senior age or were not known to the community members to whom they were expected to distribute the drugs getting selected. Lack of school attendance and seniority (>35 years) in age can be confirmed from the socio-demographic profiles presented in Table 1. As indicated by some of the opinion leaders, there was a lack of transparency in the CDDs selection process with those not fitting in the criteria getting selected. Notably not all opinion leaders are village leaders as some are political or religious leaders meaning that they may not have been involved in the CDDs selection process and were able to point out CDDs incompetency in drug distribution attributable to non-adherence of the selection criteria.

An Opinion leader thus stated:

“From what I can see, the distributors, some of them were a little old, youths who are still energetic and can walk with this hot weather and cover a bigger area should be chosen. Those old people that I saw distributing the drugs cannot do this work well. Look for those young people who can walk and do this job well.” (IDI-OL-003-Kaloleni)

*“*Conduct transparent selection of the CDDs, look into their age, choose young people, who have been to school. Ensure that their character is credible and they are known and relate well with the community members.” (IDI-OL-008 Kayafungo)

An opinion leader in Kaloleni stated that:

*“*They should engage people who have been to school. People who can reason out.” **(**IDI-OL-007- Kaloleni)

Through the CDDs FGDs, it is apparent that there are a lot of CHVs but it is the same ones that get to be selected for the CDD role causing a feeling of discrimination among those who never seem to get a chance.

The CDDs stated that all CHVs needed to be given the opportunity and that the same CHVs should not be used in every round of MDA.

A CDD stated that:

*“*Another one is that, there are a lot of CHVs, selecting the same CHVs each time there is MDA is bringing some problems to some of them. They feel discriminated. They should rotate and make sure all the CHVs are actually involved in the MDA.” *(*CDD*-*FGD-003- Kayafungo)

The other issue reported as a challenge to drugs uptake posed by the CDDs deployment was the posting of CDDs to areas where they were not known by the community members.

A CDD thus stated:

“Let them use the CHVs that the communities know because even if you take me as CHV from Mwanda village and post me to Gogoraruhe village, there will be a problem as I am not known there.*”* (CDD-FGD-004- Kaloleni)

“The first challenge is that the village which I come from is very far from the one which I was told to distribute the drugs in.” (CDD-IDI-003-Kayafungo)

An opinion leader from Kayafungo ward further stated that:

*“*If a CDD does not come from the same village, it can raise some concerns that lead to refusals, but when it is their own son or daughter, the villagers will not have any problems.” (OL-IDI-003- Kayafungo)

To address the issues related to the CDDs selection process, it is imperative for the program to ensure that the prescribed selection criteria- known and belonging to the community, able to distribute drugs to all eligible members in the allocated households, ability to read and write, keep records and trustworthiness is adhered to so that the CDDs are accepted by the community members. Importantly there is need to resolve the problem of CDDs feeling discriminated by ensuring that all CHVs are rotated accordingly so that each gets a chance to participate in MDA.

### Inadequacy of number of CDDs in comparison to the area to be covered “These villages are big with many households and can’t be served by one person”

The challenge of one CDD covering a whole village was voiced as another reason for low MDA uptake. The CDDs are expected to cover a village with an estimated maximum of 300 households each in total. In villages where the households are more than 300, the CDDs felt that the workload was too much for them given the period within which they were expected to make returns. Furthermore, the households in some villages are far apart causing a feeling of being overwhelmed as it requires the CDDs to spend a lot of time walking from one household to the next. The CDDs felt that more days should be allocated for drug distribution to enable them to cover all the households.

A CDD from Kaloleni ward stated that:

“The CDDs are also few and so they do not reach all the places. Sometimes you are given the drugs to distribute but the village is so vast and you are expected to distribute to the whole village on your own. So, it is a tedious task. Sometimes the same day you are given the drugs, you are told to start distributing the drugs. That is a half day you are expected to cover the whole village and within are short time to be done and return the records.” (CDD-FGD-003-Kaloleni)

In Kayafungo ward the participants in the CDD FGD further stated that:

*“*Another one is that the CDDs are very few. You can imagine a whole village covering like 300 households is given only a CDD to distribute the drugs. There will be problems during registration because they won’t reach all the households to register them. When they start distributing the drugs some community members are not given.” (CDD FGD- 004- Kayafungo)

*“*They should go back to how things were done before, we were 4 CDDs at that time and now we were reduced to 2 per village. They give us a day to distribute drugs to the whole village, how?” (CDD-FGD-004-Kaloleni).

Some participants in the IDIs with opinion leaders felt that there was a problem with the number of the CDDs engaged in the MDA. The few number of CDDs was raised as a reason for the low coverage and uptake. An opinion leader from Kaloleni and another from Kayafungo respectively stated that:

“In the MDA for swollen limbs and swollen genitals the problem I think is that the ones who were given the task of distributing the drugs maybe were not enough.” (OL- IDI-004-Kaloleni)

*“*These people should be more than one and given enough time to conduct awareness and distribute the drugs. “To add on to that the CDDs were very few in the field because the ones concerned with the allocation didn’t want to spend a lot of money paying the CDDs. The area to cover is large and the CDDs sent to the field are few. So if you don’t change on this expect worse results.*”* (OL-IDI-001-Kayafungo)

The heath workers also stated that the vastness of the area to be covered versus the number of CDDs was a challenge. A health worker stated that:

“In the last MDA the CDDs were very few and they were to cover the vast area, they did not cover all the places thus they didn’t get to reach all the people.” (HW- IDI-001-Kaloleni)

To address the problem of inadequacy of CDD numbers, the program implementers from national to county to ward level are called upon to look into ways of increasing the number of CDDs in villages that have more than 300 households and in those where the households are sparsely distributed so as to ensure that no eligible community members are left out during registration and drug administration.

### Inadequacy in CDDs’ training “The training is just a briefing for a few hours”

Regarding their perception of the training received, the CDDs reported that sometimes the trainings are not given at all or that they received only a few hours of briefing in the morning and were expected to conduct household registration from the afternoon of the same day. This was seen as a major shortcoming bearing in mind that the CDDs would be expected to create awareness about LF disease, fill in household registration forms and drugs tallying sheets and respond to community members’ questions during MDA. The total lack of or inadequate training results in the CDDs being unable to respond to questions raised by community members during drug distribution. A CDD from Kayafungo ward stated:

“The CDDs work is about public health, and main responsibilities are to sensitize and educate the community members, in order to realize good health practices, it is about sensitization and going door-to- door to distribute drugs. They also educate the community on use of toilets, sensitizing pregnant women on the importance of visiting the hospitals during pregnancy. They also encourage community members to discard old traditions and start living a healthy life and demystifying the causes of swollen limbs. The message they give to the community is from their knowledge of public health because there are no trainings they are given.” (CDD-FGD-003-Kayafungo)

A CDD from Kaloleni ward stated that:

*“*You know in the past we used to have trainings for CDDs like at Kaloleni and have briefings and trainings on how to use the drugs but then they stopped doing that. Today we are just contacted like you have been told, we go to Gotani village and they instruct us to go to the community and tell the people there will be MDA, register their names, and when you return from Gotani village with the records, you are given the number of drugs according to the people you registered and told to start the distribution process so, there is no training for CDDs anymore.” (CDD-IDI-004-Kaloleni)

*“*I suggest that next time the CDD should have medical knowledge and this will help them to explain the importance of the drugs.” (CDD-IDI-005-Kayafungo)

The program implementers are again called upon to improve on coordination and ensure that CDDs training is given enough time as required. Adequate and well planned training would assist in confidence building allowing the CDDs to better conduct their role for improved community drug uptake.

### Insufficiency of drugs issued to CDDs for distribution “The issue of shortage of drugs and those who can help you are far, so I urge the government to ensure that we have enough drugs”

The CDDs expressed that they experienced logistical challenges related to running out of drugs during administration and it being difficult to get more as the drugs are issued according to the registered number of household members.

A CDD stated that:

“Sometimes you get a household with more people than those registered such as with visitors, you cannot leave them out, you give them the drugs.” (CDD-FGD-004-Kayafungo)

In order to make work easier, CDDs felt that there was need to ensure that they had enough drugs especially since whenever they ran out of drugs they had to go back to the distribution points which in some cases was located very far resulting in time wastage and negatively affecting uptake.

A CDD stated that:

“There is no system to replenish the stock of drugs when they get finished and walking to and fro the facility every time the drugs are exhausted affects our target.” (CDD- FGD-001-Kaloleni)

The local health facilities together with the CHEWs need to come up with a plan of ensuring that there are no gaps in replenishing drug stock-out among the CDDs during house-to-house administration. Proper household registration ensuring that the correct numbers are captured and giving enough buffer would help address the problem of insufficiency of drugs issued to CDDs thus improving MDA uptake.

### Lack of CDDs’ supervision “There should be someone who will be oversee these CDDs, even if he won’t literally visit each house”

The CDDs further highlighted the lack of supervision by the CHEWs during MDA as a challenge to their performance indicating that the role was completely left out to them. This would imply that there was a lack of moral support with the CDDs not having supervisors to guide them on drug distribution and listen and help solve the challenges that they were encountering. From the CHEWs point of view, the reason given for failure to supervise was lack of transport to access the villages where the CDDs were, given that one CHEW was expected to supervise a minimum of 4 CDDs. A CHEW stated that:

*“*Since vehicles cannot go into villages, we usually use motorbikes and not all officers have motorbikes.” *(FGD-KLN-HW-003)*

A CDD also stated:

“You know there is no one who will come to confirm if the drugs were really given to all the eligible people. The CDDs can even lie that they gave drugs to some households while they did not. Like last year the person who came distributing the drugs in Kibao Kiche village came from Pangayambo village. Obvious he did not cover all the households and then the records say Kibao Kiche was covered.” (FGD-CDD-003- Kayafungo)

The opinion leaders further reiterated the lack of CDDs supervision as a drawback to the program and indicated that supervisors to ensure that the distribution was carried out were important. An opinion leader stated that:

*“*So, a CDD can just fill-in the details without actually going around to each village. That’s why some people don’t get the drugs. You know when they return records, the supervisors will see they attended to all the villages but no. There should be someone overseeing the whole process, like in this part of the village the CDD came, but when the supervisors visit each village they will discover CDDs did not go to all the villages. Make sure you follow closely these people to ascertain that they have actually visited all the places.*”* (OL-IDI-001-Kayafungo)

Supervision of CDDs during the drug distribution by the CHEWs needs to be well coordinated and its cost factored in the program. CHEWs need facilitation to traverse the areas and supervise the CDDs allocated to them. This would ensure that problems encountered by CDDs are resolved in a timely manner and that they, CDDs feel morally supported to conduct drug distribution.

### Motivation of the CDDs “I do this work to see people living in good health”

The CDDs reported that various aspects motivated them to participate in MDA. Some of the aspects included: seeing the community members happy, eradication of disease, recognition from the community for the good job they are doing, and the training and experience of participating in the activity.

A CDD stated that:

“I do the work first because I love it with my heart, and because I am helping the community. It’s just that, helping my community in improving their health status. Because when I help them in matters related to health then you are contributing to healthy living in the community.*”* (CDD-FGD-004-Kaloleni)

There were CDDs who mentioned that the payment they received motivated them to distribute the drugs while some stated that it was a God given opportunity as not any community member would be assigned such a role. A CDD stated that:

“At the end of the exercise we receive some payment that motivates me more.” (CDD-IDI-008 Kaloleni)

“I did the work because it assists the community I did not disagree because it’s an opportunity God gave me others desired and they didn’t get the chance but for me I was happy furthermore the work is tiresome but I did it with a passion.” (CDD-IDI-002-Kaloleni)

“To me for real I benefit from the trainings I get. I value education so much that’s why anything that will make me learn something from it I like to participate.” (CDD-IDI-003-Kayafungo)

The opinion leaders however indicated that the CDD’s payment should be improved, noting it is not really a payment but something to boost their morale and further suggested coming up with a way of recognizing the villages that have high drugs uptake would further motivate the CDDs.

*“*So even they (CDDs) when you give them something bigger, they get the moral and they will do the work.” (IDI-OL-008-Kaloleni)

“A village that has the highest number of people taking the drugs should have the CDD rewarded. It can be a small present. This will encourage many people to take the drugs too. Everyone who takes the drugs or everyone who the CDDs have visited in their homes should also be recognized. The drugs will be taken by a large percentage of people.” (IDI-OL-002-Kayafungo).

While the program is able to provide monetary incentives to the CDDs, the communities need to come up with innovative ways of motivating the drug distributors. Recognition of CDDs who distribute drugs to villages that achieve a high drug uptake as suggested by the opinion leaders is one of the ways to motivate them. Others ways include giving them adequate training and just appreciating their work through intrinsic motivation as they, CDDs feel honored to be assigned the drug distribution role.

## Discussion

This study explored implementation challenges and opportunities for improved MDA uptake based on the experiences and perceptions of the CDDs. The results have shown that there are several challenges experienced by CDDs during MDA campaign and that affect communities’ uptake of the drugs. From the perspective of the CDDs, the results also show opportunities to mitigate the challenges that could potentially improve drug uptake.

Fear of side effects coupled with myths and misconceptions about the disease cause due to inadequate health promotion activities, community sensitization and awareness creation according to the CDDs resulted in communities’ refusals to take the drugs. The community members complained of not being prepared for the MDA with essential information about the need for treatment, eligibility criteria, drugs used and potential side effects, which contributed to their refusal to take the drugs. Similar results have been reported in a study conducted in Indonesia which emphasized on the importance of filling the knowledge gap with regards to the disease as well as the MDA [20]. The importance of delivering appropriate health information to address people’s concerns and fears about the intervention, create awareness about the disease and make arrangements for the management of side effects has been reiterated in a study conducted in India [21].

From the CDDs perspective, a well-planned health promotion campaign regarding the disease and MDA that is done repeatedly throughout the year and not only during the campaign period is a step towards improving drug uptake. This study therefore calls for the NTDs Program at National Level to collaborate with county stakeholders and consider facilitating the implementation of health promotion activities that run throughout the year using combined behaviour change communication strategies. A study conducted in American Samoa highlighted the positive impact of planning awareness campaigns on persons agreeing to participate in order to avoid being infected [22].

In the current study, use of health promotion and awareness creation materials such as the pamphlets distributed to households was considered by the CDDs as an impactful way of passing the information and community members were reported to accept the drugs on seeing images of persons disfigured by LF. This study encourages continued distribution of printed awareness creation materials to individual households for improved understanding of the need to take drugs and prevent the progression to disfigurement and morbidity due to LF. Similarly, in a study conducted in Dominican Republic, community members emphasized that the use of photos of persons with lymphoedema and hydrocele during awareness creation was beneficial to drug uptake [23].

Lack of hygiene in drug handling was another challenge attributed to communities’ refusal to take the drugs based on the current study results. The community members felt that the CDDs needed to handle the drugs hygienically while dispensing them to avoid contamination. The CDDs requested to be provided with gloves or spoons by the program. The World Health Organization requires that while dispensing medicine one should count and measure the medicine carefully and guard against contamination by using clean equipment and never allowing skin contact with the medicine [24]. The Kenyan program and indeed all other programs need to come up with affordable and convenient ways of ensuring that hygiene is maintained during drug administration.

Absenteeism of community members during drug administration has also been reported as a challenge in the current study. Absenteeism could be attributed to migration due to occupation where people move to other localities for farming activities, casual labor or to urban areas for employment. Migration of persons negatively affects MDA uptake and the findings are consistent with those of a study conducted in Sierra Leone [25]. Other reasons contributing to absenteeism could be lack of awareness of distribution mode and schedules for MDA due to inadequate community sensitization activities that lack repeated messaging and reminders. Similarly, results of a study conducted in India identified the provision of information regarding distribution mode and schedule as a probable way of improving persons’ availability during MDA [26]. Designing appropriate drug delivery strategies to reach all populations though seen as a challenge by many programs has been reported to lead to sustained program uptake [27]. The current study recommends the program to consider different delivery approaches so as to reach migrant populations.

The selection process of CDDs, which gives the criteria of ability to read and write; keep records; trustworthiness; well known by the community members; and willingness to distribute drugs to all eligible persons in allocated areas which saw factors such as level of education and age considerations not being adhered to has been reported to have had a negative influence on community participation in MDA in the current study. CDDs who are advanced in age and who have never attended school at all for basic literacy are not ideal for drug distribution as the job entails traversing vast areas and they would also encounter problems with regards to awareness creation and record-keeping. The Kenyan LF program has been running for many years and there would be need to ensure proper involvement of the communities in the selection process to allow them identify the CDDs that they consider appropriate. Krentel et *al*. (2017) encourages use of social networks to help communities identify CDDs who would be able to reach a greater proportion of the population [28]. CDDs relationship and acceptability by communities has been reported to be a potential mediating factor for quality campaign and high treatment uptake [29]. The selection of CDDs through community-wide meetings is seen as a key driver of empowering local ownership of MDA and ideally increasing treatment rates but there are recommendations for exploring other methods. Selecting CDDs who belong to supportive friendship groups and who could participate in the trainings and potentially contribute to awareness creation and community mobilization and thus increase drug uptake has been suggested by Chami et *al*., 2019 [30].

The results of the current study have also shown that CDDs were deployed to distribute drugs in villages that they do not belong to. This was considered an implementation challenge as the CDDs did not know the geography of the area allocated to them and being strangers, were not well received by the community members. Similar results of a study conducted in Sri Lanka showed that drug uptake was high when the community knew their drug distributor [31] and on the flip side low acceptability of the drug distributor in India negatively affected the uptake [32]. Adherence to the recommended criteria of selection is important in improving interaction of CDDs and community members for improved drug uptake. Furthermore, the results of the current study show that community members complained that the same persons are deployed as CDDs at every round of MDA and argued that every CHV needed to get the opportunity otherwise those left out felt discriminated against an issue that was causing animosity among the CHVs. Giving all CHVs who meet the selection criteria equal opportunities in the LF program as a way of incentivizing them is important for the program’s success as the CHVs by playing the role of CDDs feel recognized and trusted by the community which is similar to findings reported by Fleming et *al*., 2016 [33].

The current study results also indicate that there was a shortage of CDDs assigned for the MDA campaign thereby negatively impacting on reaching all targeted community members. CDDs are the frontline personnel in MDA for LF programs and hence any shortages could negatively affect implementation [34]. The current study also showed that the CDDs felt pressurized by the workload based on the vastness of the area that they were expected to cover in the given period. A previous study conducted in Kenya reported that allocation of too many households to CDDs negatively affected their performance and motivation, as they found themselves overburdened with work which resulted in poor interaction with the community members and thus low drug uptake [35]. Similar studies have recommended that the program implementers consider allocating households to CDDs based on vastness the area and the sparse distribution of the population and to also consider their numerical strengths [36,37]. Results of a study conducted in Nigeria where CDDs complained of high workloads and requested for recruitment of more CDDs to support them on the job have been reported [38].

Inadequacy of drugs issued to the CDDs for distribution was another challenge to the implementation of MDA based on the current study results. The CDDs reported running out of stocks and having to spend time organizing for re-fills. Similar results related to drugs stock-out have been attributed to poor CDDs performance and low drug uptake [33]. The study by Krentel et *al*., (2017), explains how health systems’ challenges such as when supplies are inadequate for the program activities affect implementation of the program by becoming challenges of the CDDs [28]. Kamara et *al*., (2019) recommend pre-MDA registrations in areas where populations are highly mobile which are implemented correctly to avoid missing pockets of population as a way of ensuring that CDDs receive adequate supply of drugs for distributions [25]. The Kenyan NTD program is encouraged to collaborate with all relevant stakeholders to ensure that no households miss out on registration and subsequent drug distribution for successful LF campaigns.

The training of CDDs on good communication skills, the disease and its prevention have been highlighted as important for the CDDs’ acceptance by communities and also for their own motivation in conducting drug distribution [39]. Results of the current study show that the CDDs were not adequately trained to perform the role. The confidence and motivation of CDDs as a result of training have also been highlighted in the Togo program which has been highly successful [40]. Failure to adequately train and supervise the CDDs has been known to contribute to their being ineffective in carrying out their duties [33,35]. The previous study conducted in Kenya reported that a lack of supervision from health workers could be attributed to reduced CDD motivation in the program [13]. Factors to do with improper training, insufficient supervision, and confusion around responsibilities and the limited amount of time allocated for workload have been reported as demotivating factors for CDDs in policy documents systematic review and analysis [41].

### Study limitations

This study had some limitations. Firstly, the data were collected in local languages which could have resulted in loss of variations in dialect during the translation process. Secondly, there may have been introduction of recall bias for the CDDs who had distributed drugs during the 2017 MDA round and who were interviewed beyond 3 months after the campaign.

### Conclusions and recommendations

The findings of this study have shown that there are several implementation challenges experienced by the CDDs that are related to the community, the health system and the NTD program itself and that should be addressed in a more holistic way. The CDDs who are key implementers of MDA need support at the various components of the health system, the community and the NTD program for successful LF elimination campaigns. Strong collaborations are needed between the NTD program, the health system and the communities noting that community participation and ownership is a key step towards program success and sustainability. The findings are important for fast tracking the achievement of equitable access to health. Opportunities for mitigating the challenges cut across provision of information through awareness creation and health promotion that is appropriately scheduled by the NTD program. Transparent CDDs selection process by the community with support of the health personnel, adequate training and supervision need to be conducted. Consideration of the workload in order to engage sufficient human resource to meet the existing demands is necessary to prevent the CDDs from feeling overburdened and for ensuring that all community members are reached. Sufficiency of drugs issued to the CDDs and that are hygienically dispensed to the community members is important in overcoming frustrations. Intrinsic and extrinsic appreciation and motivation of CDDs are key factors for their willingness to continue serving the communities.

## Supporting information

S1 Text Appendix 1 CDDs FGD Guide. (DOCX)

S2 Text Appendix 2 CDDs IDIs Guide. (DOCX)

S3 Text Appendix 3 Opinion Leaders IDI Guide. (DOCX)

S4 Text Appendix 4 Health Workers IDI Guide. (DOCX)
