# Supplementary material for: Implementation challenges and opportunities for improved mass treatment uptake for lymphatic filariasis elimination: Perceptions and experiences of community drug distributors of coastal Kenya
Source: PLoS Negl Trop Dis. 2020 Dec 28;14(12):e0009012. doi: 10.1371/journal.pntd.0009012 (PMC7793263; doi:10.1371/journal.pntd.0009012)
Supplement: S1 Text — (DOCX) [file pntd.0009012.s001.docx]

**Appendix 1: Focus Group Discussions with community drug distributors**

| Instructions:   - This form should be used for FGDs for the community drug distributors. - If the participants refuse to answer a question, circle the number of the question and do not mark any answers for that question. - After obtaining informed consent, read the following instructions to the participants: |
| --- |
| **“I am going to ask you questions about the Filariasis control program, so as to collect information about your knowledge of LF and opinion of MDAs, the challenges experienced during implementation and your thoughts on opportunities for improved MDA uptake. Please answer the questions as honestly as you can remember. Your information which will be tape recorded will be kept private and this form will not have your name anywhere. If you have any questions or do not understand what I am asking you at any time, please ask for clarification. Some questions may prove embarrassing to you.**  **Please remember that you do not have to answer any questions that you do not want to answer and you may discontinue the discussion at any time. Do you have any questions before we begin?”** |

**Socio-Demographic Characteristics**

| Sub-county: | | | Moderator: | | |
| --- | --- | --- | --- | --- | --- |
| Ward: | | | Note taker: | | |
| Date of FGD: | | | Time start: | | |
| Location of FGD: | | | Time stop: | | |
| Participants at start | | | Debrief notes | | |
| Participants at stop | | |  |  |  |
| **Participant** | **Age** | **Sex** | **Level of Ed.** | **Religion** | **Occupation** |
| 1 |  |  |  |  |  |
| 2 |  |  |  |  |  |
| 3 |  |  |  |  |  |
| 4 |  |  |  |  |  |
| 5 |  |  |  |  |  |
| 6 |  |  |  |  |  |
| 7 |  |  |  |  |  |
| 8 |  |  |  |  |  |
| 9 |  |  |  |  |  |
| 10 |  |  |  |  |  |

**Focus Group Questions**

**Selection process**

1. How were you selected as a drug distributor?

Probe for reasons for accepting

**Training**

1. Did you undergo any training for the drug distribution?

Probe for who trained you, the content, duration and venue

**Collection of drugs**

1. How many drugs did you require?

Probe for the number received, where collected, problems experienced during collection and nature of problems experienced if any

**Mode of drug distribution**

1. How did you go about the drug distribution? (Probe for the number of households supposed to cover, those covered, number of days used etc.)

- How do you know that all the people were given drugs and consumed them?
- Were all sections of the community covered by the drug distribution?
- Did anybody help you in the distribution? (Probe for who and type of assistance given)
- Did you face any difficulty? If yes, describe.
- How did you resolve the difficulties?
- What suggestions do you have for improving the drug distribution?
- Would you be willing to take part in the distribution next time? If yes, or no, elicit reasons.

**Management of side effects**

5. Did people report any problems after taking the drugs? Probe for

- What problems specifically?
- How were these problems managed?
- Who managed these problems?

**Record maintenance**

6. Did you maintain any records regarding the drug distribution?

- If no, elicit reasons.
- If yes, can you show us some of these records? (Interviewer to take notes on type of records kept).
- Did you face any problems in making these records? If yes, what problems?
- How did you resolve them?

**Information, Education and Communication (IEC)**

7.Was the community that you distributed drugs to informed about the MDA?

- If yes, how did they get informed?
- Were you involved in informing them?
- Do you think that the materials used to inform the community members about MDA were adequate for their understanding?
- If not, what suggestions can you give for improving the materials used for awareness creation?

**Incentives**

8.What kind of support do you receive from the community in your role as a CDD? Probe

- Financial
- Moral
- Food
- Nothing
- Others, specify ________________________________

9. If you received moral support from the community, whom did you receive it from? Probe

- Community leader
- District LF Coordinator
- Community LF Coordinator
- Health Committee
- Others, specify _______________________________________

10. Who would you say has been most supportive? Probe for what they did

11. Who would you say has been least supportive? Probe for what they had in their powers to do but failed to do to support?

12. Is the two-day period of MDA a satisfactory length of time for distribution of the drugs to your community? Probe for reason given for the answer if yes or if no

**THANK YOU VERY MUCH FOR YOUR COOPERATION**
