# Supplementary material for: Implementation challenges and opportunities for improved mass treatment uptake for lymphatic filariasis elimination: Perceptions and experiences of community drug distributors of coastal Kenya
Source: PLoS Negl Trop Dis. 2020 Dec 28;14(12):e0009012. doi: 10.1371/journal.pntd.0009012 (PMC7793263; doi:10.1371/journal.pntd.0009012)
Supplement: S2 Text — (DOCX) [file pntd.0009012.s002.docx]

**Appendix 2: In-depth Interview with community drug distributors**

ID: ________________

Date: ___________________

Sub-County: ______________________

Ward: _______________

Name of interviewer __________________________________

**Socio demographic characteristics**

Sex:

Age in years

Designation:

Highest Education Level:

Religion:

**Selection process**

1. How were you selected as a drug distributor?
2. Are there any special reasons as to why you agreed to distribute drugs?

**Training**

1. Did you undergo any training for the drug distribution?
2. Who trained you?
3. What were the content, duration, and place of training?

**Collection of drugs**

1. How many drugs did you require?
2. How many drugs did you get?
3. From where did you collect the drugs?
4. Did you experience any problems in collecting the drugs?

If yes, describe the nature of the problems.

**Mode of drug distribution**

10. How did you go about the drug distribution? (Probe for the number of households supposed to cover, those covered, number of days used etc.)

11. How do you know that all the people were given drugs and consumed them?

12. Were all sections of the community covered by the drug distribution?

13. Did anybody help you in the distribution? (Probe for who and type of assistance given)

14. Did you face any difficulty? If yes, describe.

15. How did you resolve the difficulties?

16. What suggestions do you have for improving the drug distribution?

17. Would you be willing to take part in the distribution next time? If yes, or no, elicit reasons.

**Management of side effects**

18. Did people report any problems after taking the drugs?

19. What problems specifically?

20. How were these problems managed?

20a. Who managed these problems?

**Record maintenance**

21. Did you maintain any records regarding the drug distribution?

If no, elicit reasons.

22. If yes, can you show us some of these records? (Interviewer to take notes on type of records kept).

23. Did you face any problems in making these records? If yes, what problems?

24. How did you resolve them?

**Information, Education and Communication (IEC)**

25a.Was the community that you distributed drugs to informed about the MDA?

b. If yes, how did they get informed?

c. Were you involved in informing them?

d. Do you think that the materials used to inform the community members about MDA were adequate for their understanding?

e. If not, what suggestions can you give for improving the materials used for awareness creation?

**Incentives**

26a.What kind of support do you receive from the community in your role as a CDD? (Tick)

1. Financial
2. Moral
3. Food
4. Nothing
5. Others, specify ________________________________

26b. If you received moral support from the community, whom did you receive it from? (Tick)

1. Community leader
2. District LF Coordinator
3. Community LF Coordinator
4. Health Committee
5. Others, specify _______________________________________

26c. Who would you say has been most supportive? ______________________________

26d. What did they do in particular? __________________________________________ ________________________________________________________________________________________________________________________________________________

26e. Who would you say has been least supportive? ______________________________

26f. What do you think they had in their powers to do but failed to do so in your support? ________________________________________________________________________________________________________________________________________________

27. Is the two-day period of MDA a satisfactory length of time for distribution of the drugs to your community? (Tick)

1. Yes
2. No

28. If no, what length of MDA do you think is satisfactory? ______________days

29. If yes, what makes you say so? ________________________________________

**THANK YOU VERY MUCH FOR YOUR COOPERATION**
