# Supplementary material for: Implementation challenges and opportunities for improved mass treatment uptake for lymphatic filariasis elimination: Perceptions and experiences of community drug distributors of coastal Kenya
Source: PLoS Negl Trop Dis. 2020 Dec 28;14(12):e0009012. doi: 10.1371/journal.pntd.0009012 (PMC7793263; doi:10.1371/journal.pntd.0009012)
Supplement: S3 Text — (DOCX) [file pntd.0009012.s003.docx]

**Appendix 3: In-depth Interviews with opinion leaders**

| Instructions:   - This form should be used for in-depth interviews with the opinion leaders. - If the participants refuse to answer a question, circle the number of the question and do not mark any answers for that question. - After obtaining informed consent, read the following instructions to the participants: |
| --- |
| **“I am going to ask you questions about the Filariasis control program, so as to collect information about your knowledge of LF and opinion of MDAs, your community members’ willingness to participate in the program and their preferences for being reached during the campaign, barriers affecting the programme and existing opportunities and outlets that could be used to reach the communities during MDA. Please answer the questions as honestly as you can. Your information which I will tape record will be kept private and this form will not have your name anywhere. All the information will be kept confidential until the conclusion of the study when it will be destroyed. If you have any questions or do not understand what I am asking you at any time, please ask for clarification. Some questions may prove embarrassing to you.**  **Please remember that you do not have to answer any questions that you do not want to answer and you may discontinue the discussion at any time. Do you have any questions before we begin?”** |

**Fact sheet**

ID_______________________________________

Time_____________________________________

Date______________________________________

Sub-county_____________________________________

Ward____________________________________

**Socio-Demographic Characteristics**

1. Sex (Tick) Male (1) Female (2)

2. Age in Years _____________________

3. Marital Status (Tick)

Single (1)

Currently Married (2) (Tick) Polygamous (1) Monogamous ( 2 )

Divorced (3)

Widow/ widower (4)

4. Religion (Tick)

Christian (1)

Islam (2)

Non-practicing (3)

Others, specify (4) __________________________

**Socio-economic characteristics**

5. Level of Education (Tick)

Never attended school (1)

Did not complete primary school (2)

Completed primary school but did not complete secondary school (3)

Completed secondary school (4)

Further studies after secondary school (5)

Others, specify (6) ___________________________

6. Main occupation (Tick)

Farmer (1)

Small business (kiosk, kibanda) (2)

Big business (shop) (3)

Housewife (4)

Salaried worker (teacher, police, chief) (5)

Fisherman (6)

Casual laborer (7)

Others, specify (8) ________________________

**Actual Interview**

| **Questions** | **Observations (record non-verbal behaviors)** |
| --- | --- |
| 1. Could you tell me about the MDA for swollen limbs and swollen genitals in your community? Probe for |  |
| 1. Who does it? |  |
| 1. When is it done? |  |
| 1. How is it done? |  |
| 1. Why is it done? |  |
| 2. How do people in your community know about MDA for swollen limbs and swollen genitals? Probe for |  |
| 1. Adequacy of information |  |
| 1. Enough time given to understand the information |  |
| 1. Too much or too little information given |  |
| 1. Period between that which the information is given and the drugs are distributed is too soon or too long 2. Preferred modes of awareness creation |  |
| 3. How was the participation of your community members in the last MDA for swollen limbs and swollen genitals? Probe for |  |
| 1. Any problems or barriers |  |
| 1. Any facilitation |  |
| 4. What would you say about?   1. The present way of drug distribution |  |
| 1. The current drug distributors |  |
| 5. How can drug distribution in your community be improved? Probe about  Method of distribution  Distributors  Duration of distribution |  |

**THANK YOU VERY MUCH FOR YOUR COOPERATION**

**Post Interview comment**

| **In this part of the interview the interviewer should write notes that detail his/her feelings, interpretations and other comments. This should be done immediately after conducting the in-depth interview** |
| --- |
