# Supplementary material for: Implementation challenges and opportunities for improved mass treatment uptake for lymphatic filariasis elimination: Perceptions and experiences of community drug distributors of coastal Kenya
Source: PLoS Negl Trop Dis. 2020 Dec 28;14(12):e0009012. doi: 10.1371/journal.pntd.0009012 (PMC7793263; doi:10.1371/journal.pntd.0009012)
Supplement: S4 Text — (DOCX) [file pntd.0009012.s004.docx]

**Appendix 4: In-depth Interview with health workers**

ID: _________________

Name of Ward: _____________

Date: __________________

Type of Health Facility:

Sex of respondent:

Designation:

Name of interviewer: _____________________________

**Role of the health worker**

1. What were the activities assigned to you in the drug distribution programme in your area?
2. Who assigned these activities to you?
3. Elaborate on what activities you actually carried out in this area.
4. Did you undergo any training for the assigned role before the drug distribution?
5. Could you tell us the details of training?
6. Was the training adequate to undertake the job assigned to you for the programme? If not, why?

**Information, Education and Communication (IEC)**

1. Describe the efforts made by you to educate the community on mass drug distribution

8a. What were the issues/topics covered during the IEC efforts? Probe whether any

effort was made in informing the community on the side effects, describe the

message conveyed?

8b. Who designed the messages?

9a. What were the ways of communicating the information to the community?

9b. How did you sensitize the community on mass treatment for LF elimination?

1. How many times did you have to visit the community?
2. Did you face any problems in sensitizing the community on mass treatment for LF elimination? If yes, explain

**Drug distribution**

1. Could you describe how the drug was collected and where it was stored before distribution?
2. Was any problem faced in the collection and storage? If yes, please explain the nature of the problem and how it was sorted out?
3. How many drug distributors were involved from this area?
4. For how many days was the drug distributed in your area?
5. Was the drug given at any particular time of the day, if so why?
6. Did all eligible persons in the area receive the drug?
7. If not, which section of the area got left out and why?

**Side effects**

1. Did you come across people with side effects following drug distribution?
2. After how many days of getting the side effect did people come to you for help?
3. Explain the nature of side effects?
4. How were the side effects managed?
5. Did you play any role in the management of side effects? If yes, explain

**Record maintenance**

1. Who maintained the records on drug distribution?
2. If yes, what details were recorded?

**Suggestions for the improvement of Drug Distribution**

1. What did you feel about the implementation of mass drug administration in the community?
2. What were the problems faced in mass drug distribution?
3. Do you have any suggestions for the improvement of mass drug distribution?
4. What were the reactions of the community to the present drug distribution method?

**THANK YOU VERY MUCH FOR YOUR COOPERATION**
